# Dr. Frederick Holme Wiggin

**Published:** 1910-12

**Authors:** 


Dr. Frederick Holme Wiggin, of New York, died at Atlantic City, October 28, 1910, aged 56 years. He graduated at Bellevue Hospital Medical College in 1877. He was a prominent member of the State Medical Association during its existence, being at one time its president.

